# Redesigning EWOD Interconnections: Inkjet-Printed PEDOT:PSS Electrodes with Enhanced Pad Access

**DOI:** 10.1021/acsomega.6c01956

**Published:** 2026-06-02

**Authors:** Eli Nadia Abdul Latip, Loic Coudron, Ian Munro, Etelka Chung, Lanka Weerasiri, Ian Johnston, Christabel Tan

**Affiliations:** † School of Mechanical Engineering, 54703 Universiti Teknologi MARA, Shah Alam 40450, Malaysia; ‡ Biodetection Technologies Hub, Department of Engineering, School of Physics, Engineering and Computer Science, 3769 University of Hertfordshire, Hatfield AL10 9AB, U.K.

## Abstract

EWOD fabrication is typically costly, requiring specialist microfabrication facilities, equipment and techniques like physical vapour deposition. Printed electronics methods like inkjet printing offer cheaper and faster alternatives to typical fabrication methods, and conductive polymers like PEDOT:PSS offer advantages in flexibility, cost, and transparency. We report a low-cost fabrication approach and characterise its performance against standard ITO-glass electrodes. Ink-on-paper electrodes and patterned-on-glass electrodes are restricted to a single plane, limiting the number of electrically independent electrodes on a single conductive layer. Our design and fabrication approach, employing inkjet printing and double-sided patterning, overcomes topological constraints, addressing both connectivity and cost. PEDOT:PSS is highly compatible with inkjet printing technology, and a single pass of PEDOT:PSS printing was sufficient to achieve the desired conductivity, offering a cost-effective solution for fabricating large, multilevel, flexible EWOD electrode arrays.

## Graphical abstract

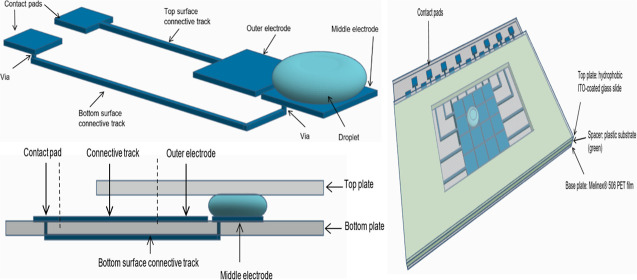

## Introduction

Digital microfluidics (DMF) has emerged over the last two decades as a robust technology capable of controlling the precise movement of multiple individual liquid droplets at microscale.^1^ Droplet movement is achieved by taking advantage of the electrowetting-on-dielectric (EWOD) effect, which enables actuation of the triple solid–air–liquid interface of droplets by electrostatic energy.^2^ An electrowetting-on-dielectric (EWOD) system typically comprises a base plate consisting of a substrate patterned with individually addressable control electrodes.^3^ These electrodes are insulated using a dielectric layer, which generates localised electric fields upon the application of a voltage across it.^4^ Compared with continuous-flow devices, DMF devices offer superior compartmentalisation, reduced cross-contamination, and lower reagent consumption. Due to its open architecture, where no physical features such as microchannels, pumps or valves are necessary, DMF technology offers significant design flexibility and ease of integration. Consequently, EWOD-based systems have been employed in various applications such as lab-on-a-chip,^5–7^ electronic cooling systems,^8–10^ and particle sampling and manipulation applications.^11–13^

Despite the widespread use of EWOD-based DMF devices, several technical and manufacturing limitations persist. A major constraint lies in the limited number of electrically addressable electrodes, which in turn restricts devices to relatively small electrode arrays.^14,15^ Traditionally, conductive tracks connecting inner electrodes to their contact pads are routed through the narrow interelectrode gaps between outer electrodes.^16^ However, the limited spacing, generally less than 150 μm, permits the routing of only a single conductive track between two adjacent electrodes, depending on the patterning technique employed.^17^ Expanding the electrode array is highly desirable, as it increases the functional area of the device, enables multiple parallel assays, and thereby enhances the overall throughput.

A popular approach for scaling up EWOD arrays involves leveraging semiconductor fabrication technologies such as complementary metal-oxide-semiconductor (CMOS),^14^ printed circuit board (PCB) substrate,^16^ and thin-film transistor (TFT) backplanes.^18^ Li et al.^14^ utilised CMOS-compatible fabrication to develop a 5 × 8 electrode array using a multilevel metallisation technique, wherein a two-layer aluminum structure facilitated the routing of electrical connections. Gong and Kim^16^ proposed a multilayer PCB approach, stacking four PCB layers with copper-plated vias to connect inner electrodes to their respective contact pads. Hadwen et al.^18^ employed thin-film transistor (TFT) backplanes, similar to those in liquid crystal displays, to fabricate a 64 × 64 electrode array with 210 μm elements. Despite their practicality, these semiconductor-based techniques are often cost-prohibitive due to their dependence on clean-room facilities and specialised materials^17,19^ and may also involve proprietary fabrication processes, limiting accessibility. The PCB method also required postprocessing, including wet etching and electrode repatterning, to reduce the height of interelectrode trenches and consequently reducing the actuation voltage from 500 to 100 V.^16^ In contrast, Jafry et al.^20^ demonstrated a cost-effective alternative using electrohydrodynamic jet printing to pattern silver nanoparticles and nanowires on a cellulose paper substrate. The silver ink wicked through the paper substrate, enabling dual-sided patterning and facilitating electrode interconnections. Nonetheless, the relatively large diameter (1.2 mm) of the silver connecting dots and the minimum 200 μm interelectrode gap constrained the electrode size and necessitated the use of larger droplets.

Fabrication of EWOD devices is often hindered by high costs associated with advanced equipment and methods such as physical vapour deposition and photolithography.^21–23^ To address this, several studies have explored alternative approaches rooted in printed electronics, which offer faster, simpler, and more economical fabrication. Techniques such as inkjet printing,^24–29^ laser printing,^17^ ballpoint pen printing,^30^ electrohydrodynamic jet printing,^20^ screen printing,^31^ microcontact printing,^32^ and spray painting^33^ have been investigated. Inkjet printing, in particular, has shown strong potential due to its scalability, high throughput, minimal material waste, and adaptability to existing facilities.^24,25,31^ It has previously been employed in organic electronics to deposit conductive layers for organic light-emitting diodes (OLEDs) and TFTs.^34,35^ Several groups have successfully used inkjet printing to fabricate EWOD electrodes on substrates such as polyethene terephthalate (PET) and paper. Wheeler et al.^22–24^ demonstrated silver nanoparticle ink printed on both substrates using both commercial (Epson C88+) and lab-grade (Fujifilm Dimatix DMP-2800) printers, enabling cost-effective point-of-care diagnostics in remote settings. EWOD devices printed using carbon nanotube ink on photo paper have also been demonstrated using low-cost desktop printers.^27–29^ Alternatively, methods such as ballpoint-pen-based silver nanoparticle deposition using digital plotters,^30^ graphite spray painting with laser-cut stencils,^33^ and screen printing using commercial carbon-based conductive inks^31^ have also been successfully demonstrated. However, EWOD devices manufactured via the inkjet printing method remain the most reliable in terms of EWOD actuation performance.^25^ Other printing methods that have been explored include laser-printed masks for PCB etching^17^ and microcontact printing using PDMS stamps.^32^ These other methods, however, are still highly dependent on photolithographic techniques to produce the electrode patterning tools.

The selection of electrode material plays a critical role in EWOD device performance. Conventional EWOD devices typically employ metallic conductors such as chromium, gold, aluminum, copper, or platinum,^14,22,23,36,37^ however, conductive polymers like PEDOT:PSS have emerged as promising alternatives, offering advantages in flexibility, processability and compatibility with low-cost fabrication techniques. PEDOT:PSS, a polymer blend of poly­(3,4-ethylenedioxythiophene) and polystyrenesulfonate, has been widely adopted in printed electronics due to its flexibility, ease of processing, low cost, and optical transparency^38–40^ offering a combination of electrical, mechanical and processing properties that make it particularly suitable as an alternative material for next-generation EWOD devices. Importantly, PEDOT:PSS can be deposited using additive manufacturing techniques such as inkjet printing, eliminating the need for vacuum-based metallisation and photolithographic patterning. Since its first use in printed transistors,^41^ PEDOT:PSS have since been employed in the manufacture of Organic Light-Emitting Diodes (OLEDs),^34,35^ photovoltaic cells,^35,42^ TFTs,^41^ and pH sensors.^38^ PEDOT:PSS has been demonstrated to be successfully deposited on substrates including glass, polyether sulfone, PET, polyethene naphthalene (PEN), and indium tin oxide (ITO)-coated PET using inkjet printing techniques and rapid prototyping soft-lithographic techniques like PDMS mold transfer.^34,35,38,39,41–43^

In the context of EWOD devices, PEDOT:PSS offers several advantages over conventional metallic electrodes as it not only lowers material costs compared to metals like silver and chrome,^40^ but also offers improved optical detection due to its transparency, facilitating integration with optical detection systems. Most EWOD devices, whether ink-on-paper or metallic patterned-on-glass, employ a single conductive layer, which inherently limits the number of addressable electrodes. To address this limitation, the present study introduces a novel low-cost, double-sided inkjet printing strategy using a Fujifilm Dimatix DMP-2850 printer to fabricate a 4 × 4 PEDOT:PSS electrode array. The profile view of the parallel-plate device’s base plate is shown in Figure 1. This fabrication strategy enables electrical interconnections between centrally located electrodes and corresponding contact pads while maintaining a simple, lithography-free process. This strategy utilises vertical interconnects, vias, to provide electrical connectivity between multiple substrates. This proposed method not only enhances device scalability and routing flexibility while reducing fabrication costs, but also overcomes the common limitations associated with traditional cleanroom-dependent techniques and device complexity.

**Figure 1:**
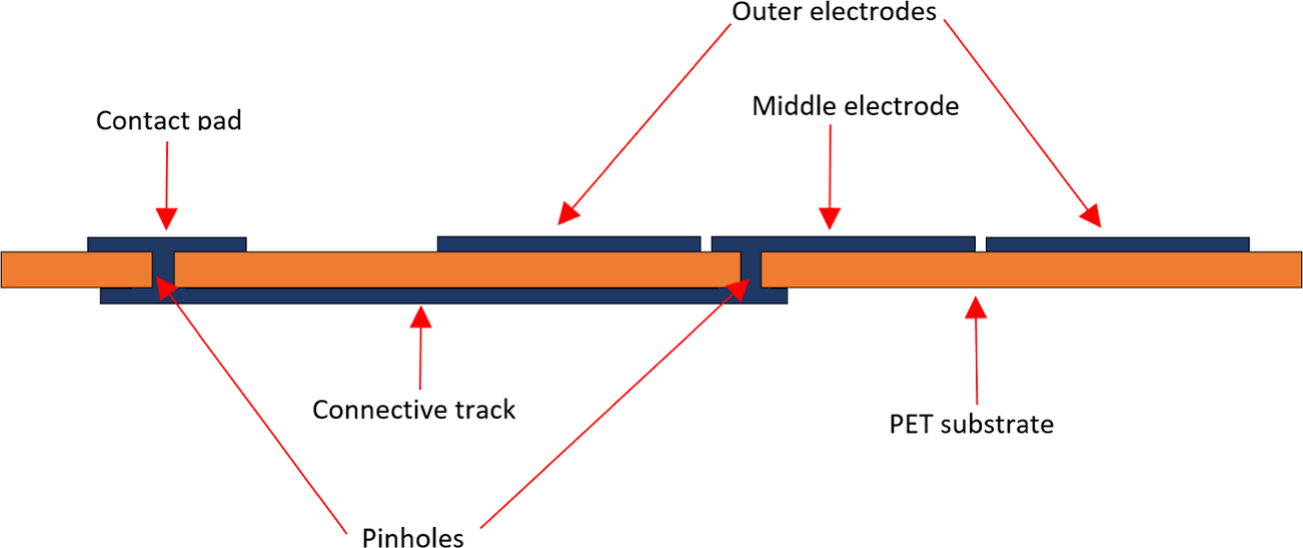
Profile view of the simplified 3D 4 × 4 electrode array device’s base plate structure. The dark blue component represents the inkjet-printed PEDOT:PSS ink, while the brown component is the polymer PET substrate. Both sides of the substrate are inkjet printed with the conductive ink to produce the connective tracks between the middle electrodes and the contact pads. Pinholes/vias are laser-cut into the middle electrodes and their respective contact pads to connect between the top-surface electrodes and the contact pads with the bottom surface tracks.

## Materials and Methods

### Electrode Array Design

The design of the 4 × 4 control electrode array is depicted in Figure 2. The first and second images show the conductive patterns printed on the top and bottom surfaces of the substrate, respectively. As both sides of the substrate are used for electrode patterning, precise alignment between the top and bottom layers is essential. Alignment markers, consisting of two squares at the top-left and top-right corners of each pattern, are used to position the substrate accurately on the printer platen prior to each printing pass. To assist with alignment, a white A4 sheet printed with identical square markers is taped onto the platen, allowing the markers on the substrate to be visually matched and aligned with those on the paper. The third image in Figure 2 illustrates the location of the laser-cut vias, which are positioned using the same alignment strategy. The final image presents a superimposed view of the top and bottom patterns along with the via locations, demonstrating their relative alignment. The nominal track width is 150 μm, and the nominal interelectrode gap is 180 μm. Two electrode sizes were designed for fabrication: 1.7 mm and 2.0 mm. Here, the term nominal size refers to the intended dimensions specified in the CAD design files, not the actual printed dimensions obtained after fabrication. The patterns were drawn using Adobe Illustrator, then converted into bitmap images with 847 dpi resolution before being transferred to the Fujifilm Dimatix DMP-2850 printer.

**Figure 2:**
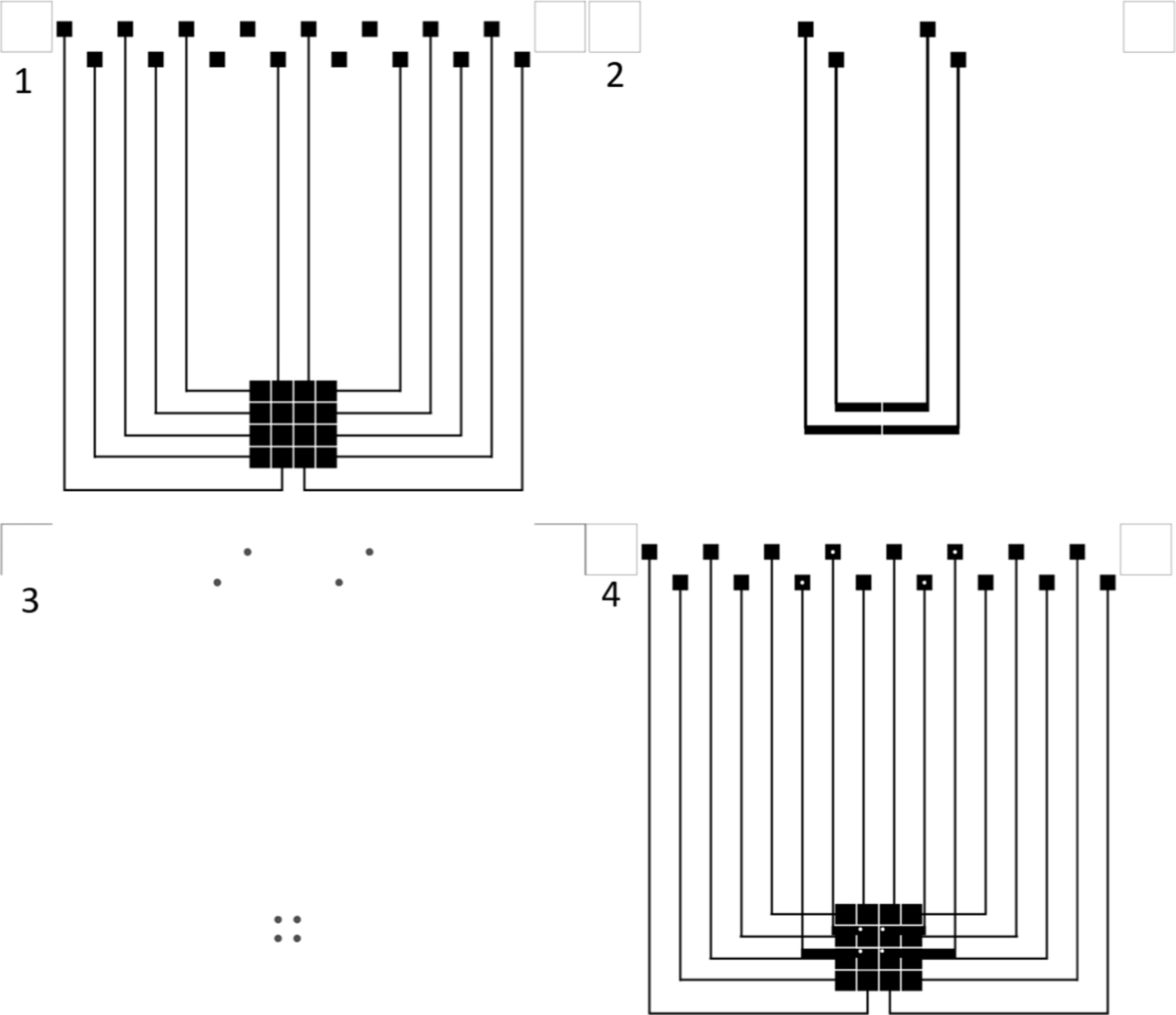
Design of the 4 × 4 electrode array. The first and second images are the top and bottom patterns, respectively. The squares at the top corners in each image are the markers for alignment on the printer platen. The third image shows the location of the laser-cut vias. The via size is exaggerated here for illustration purposes, wherein the nominal drawing size is 10 μm (the smallest possible dot size for the laser cutter software). The last image is the superposition of the top, bottom, and the laser-cut vias images.

### Vertical Interconnect (Vias) Fabrication

The presence of large particles or surface heterogeneity on the actuating surface of an EWOD device can hinder droplet motion.^44^ To mitigate this, the vias must be minimised in size, small enough to avoid interfering with droplet movement, yet sufficiently large to allow conductive ink to pass through, forming a reliable electrical connection. The via structures were designed in Adobe Illustrator using the minimum achievable dot size (0.01 mm) and subsequently exported to the laser cutter for pattern generation. Depth and diameter were controlled by adjusting two laser parameters: power and cutting speed. The smallest via diameter achieved was 105 μm, produced using 10% laser power and 100% speed. In general, the via diameter decreased with increasing laser cutting speed and decreasing laser power; smaller features being produced at higher scan speeds and lower power levels. However, at lower power settings (≤10%) and maximum scan speeds, the laser energy was insufficient to fully ablate the substrate, yielding partially formed or blind vias. For the final device, which successfully transports droplets across the middle electrodes, a laser setting of 15% power and 100% speed was used, yielding an average via diameter of 137 μm. Besides via diameter, the spatial placement of the via relative to the electrode area was found to affect actuation behaviour. Two configurations were examined to assess their influence on droplet dynamics: a centrally positioned via and a top-left-corner placement. Comparative experiments were conducted to evaluate droplet mobility, actuation uniformity, and repeatability, to enable the selection of the configuration that ensured consistent device performance.

### Conductive Inks and Polymer Substrate

Two conductive inks, PEDOT:PSS (Sigma-Aldrich, St. Louis, US) and silver ink (ANP Co. Ltd., Sejong, South Korea) were employed for implementing the 4 × 4 electrode array and the performance using the vias. Based on manufacturer specifications, PEDOT:PSS has a surface tension of 31–34 mN·m^–1^ and viscosity of 7–12 mPa·s, while the silver ink has a surface tension of 35–38 mN·m^–1^ and viscosity of 10–17 mPa·s. Initial trials showed that the silver ink failed to form a contiguous conductive path along the via walls, despite nine successive printing passes (four top, five bottom), with only two of the four middle electrodes successfully connected. In contrast, PEDOT:PSS achieved full connectivity of all four middle electrodes with just one pass per side. A Melinex 506 PET film (HiFi Industrial Film, UK) was used as the substrate. Following printing, the PEDOT:PSS layer was annealed on a hot plate at 110 °C for 5 min.

### Fujifilm Dimatix DMP-2850 Material Printer

A materials printer (Fujifilm Dimatix DMP-2850, Inc., Santa Clara, US) was used to pattern the control electrodes of the EWOD device. This printer uses a piezoelectric drop-on-demand printhead and allows three-axis cartridge movement, although printing occurs only in the *x* and *y* directions, with the substrate remaining stationary on the platen. Print resolution is controlled by adjusting the cartridge mounting angle, which sets the drop spacing. The highest achievable resolution is 5080 dpi, corresponding to a 5 μm drop spacing. For each ink type, several parameters must be optimised to achieve reliable droplet ejection, including firing voltage, meniscus pressure, cartridge temperature, jetting frequency, and the jetting waveform, which is the voltage signal applied to the piezoelectric (PZT) transducer that controls ink droplet formation. The printing height was set to 1 mm, and the meniscus was set to 3.5 in., and the firing voltages ranged from 18 to 25 V for the PEDOT:PSS ink. Printing was done at ambient conditions. To enhance ink-substrate adhesion, the PET substrate was exposed to UV/ozone treatment (Novascan PSD, Iowa, US) for 15 min prior to printing. This process increases the surface energy and improves droplet wetting ability, thereby improving the coalescence between ink dots. A maximum jetting frequency of 5 kHz was used, in accordance with the manufacturer’s recommendations for high-resolution printing, such as EWOD electrode fabrication. To further improve printing precision and edge definition, a single nozzle was used, as reducing the number of active nozzles is known to produce higher-resolution prints with better droplet placement accuracy, thus yielding finer feature resolution.^45^

### Fabrication and Assembly of the 4 × 4 Electrode Array Device

Figure 3 shows the process flow of the creation of the 4 × 4 electrode array base plate. It begins by laser cutting the vias, including the square markers, into the substrate using a laser cutter (Trotec Speedy 300, Marchtrenk, Austria). The substrate top surface is then cleaned using compressed air to remove any dust and large particles before being treated with UV/ozone cleaner (Novascan PSD, Iowa, US) for 15 min.

**Figure 3:**
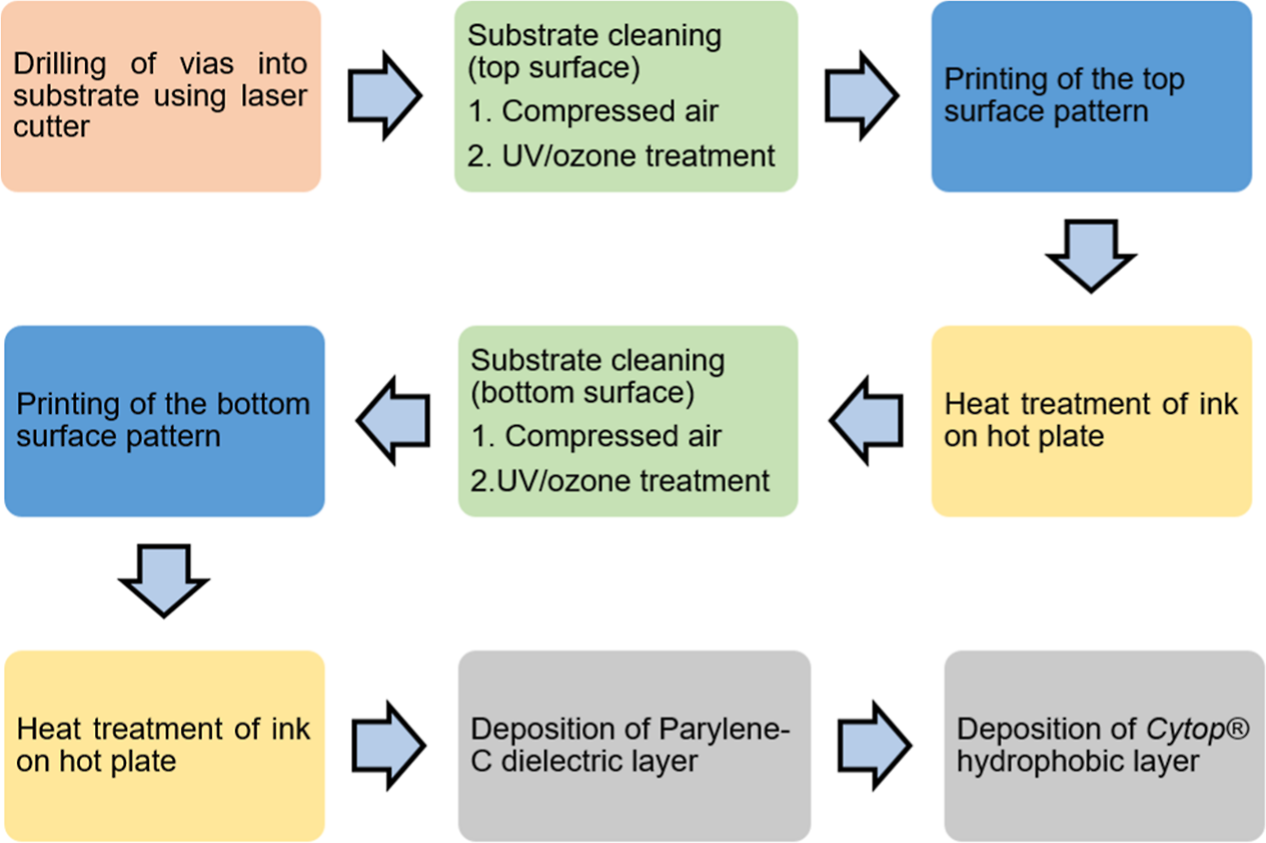
Process flow of the 4 × 4 electrode array device base plate fabrication.

Prior to starting the printing process, a white A4 paper displaying the printed square markers is taped to the printer platen. This is used as a guide to position the substrate on the platen for each printing pass by aligning the laser-cut square markers with the ones on the paper. The top pattern is printed onto the substrate, followed by a heat treatment on the hot plate at 110 °C for 5 min. Before the start of the bottom pattern printing, the bottom surface of the substrate is cleaned and treated as previously with compressed air and UV/ozone. The substrate is then heat-treated at 110 °C for 5 min after the printing is completed. Parylene-C (Specialty Coating Systems) using Parylene Deposition System (SCS Labcoter 2, Indianapolis, US) as the dielectric layer and Cytop (Asahi Glass Co., Ltd.) as the hydrophobic layer, is then deposited. The thickness of the dielectric layer was approximately 6.0 μm.

The printed base plate was assembled with a grounded cover plate made of an ITO-coated glass slide to form a parallel-plate device. A layer of Cytop was deposited on top of the ITO-coated glass slide as the hydrophobic layer. The gaps between the base and cover plates were 380 μm for the 1.7 mm electrode and 130 μm for the 2.0 mm electrode, respectively, using plastic substrates as the spacer. Different thicknesses of spacers were used for the different electrode sizes to keep the droplet volume constant. A custom-made poly­(methyl methacrylate) (PMMA) frame was employed to secure the EWOD plates in place. The setup is shown in Figure 4. Spring-loaded pins were used to connect the base plate with USB-powered compact electronics. The electronics were able to independently control all 16 electrodes using a 1 kHz sine wave with a voltage that can be adjusted up to 225 *V*_RMS_.

**Figure 4:**
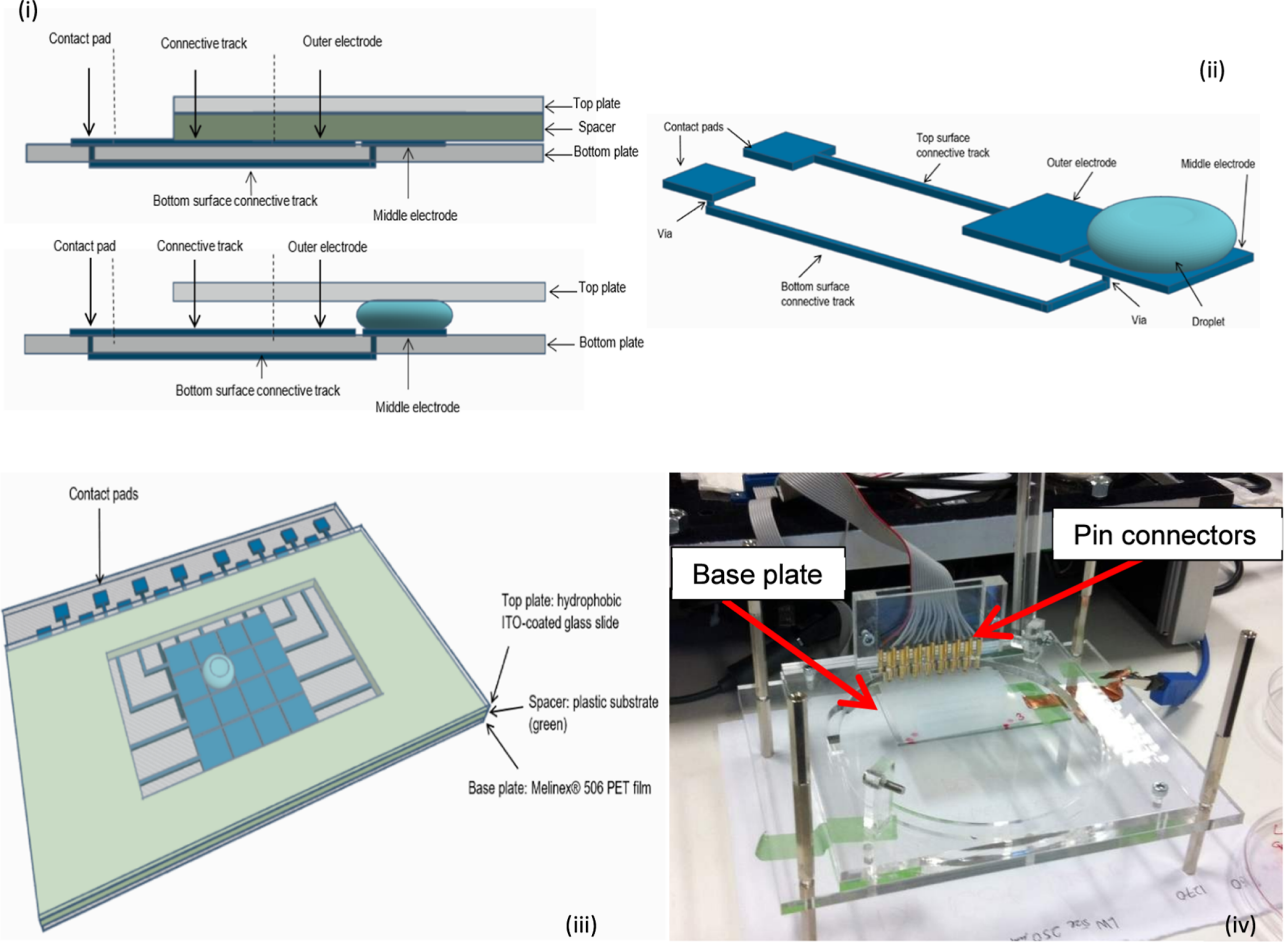
(i) Cross sections of the PEDOT:PSS 4 × 4 device, (top) with spacer (bottom) with spacer removed. (ii) Printed PEDOT:PSS tracks and electrodes with the substrate, spacer, and top plate removed. (iii) Simplified top view of the 3D 4 × 4 PEDOT:PSS device. (iv) The PMMA frame used to hold the EWOD device in place. Only the base plate is mounted in this image, and it is connected to the control electronics (not shown here) by 16 spring-loaded pin connectors (Photographs courtesy of E. Nadia. Copyright 2025).

### Contact Angle Measurement

The static contact angle of 10 μL of DI water on the surface of two types of electrode material, PEDOT:PSS-on-PET and ITO-on-glass was measured to evaluate their electrowetting property. Both types of electrodes were deposited with the same thicknesses of Parylene-C and Cytop as the dielectric and hydrophobic materials, respectively. The measurement was made using a Theta Lite optical tensiometer (Biolin Scientific, Gothenburg, Sweden) with the droplet images recorded at 1.3 frames per second (fps) and were analysed by One Attension software (Theta Lite system’s software).

### Droplet Transportation Performance Measurement

Five μL of deionised (DI) water was used to evaluate droplet transport in parallel-plate EWOD devices. The performance was compared with a chrome-on-glass plate,.^5^ The droplet movement in the 4 × 4 array device was recorded with a Canon PowerShot camera (Canon, Tokyo, Japan) at 240 fps. The video was analysed using image analysis software (Tracker, Video Analysis and Modeling Tool, California, US).

## Results and Discussion

### Inkjet-Printed PEDOT:PSS Electrodes Characterization

Table 1 reports the characteristics of the inkjet-printed PEDOT:PSS electrodes in the 4 × 4 array device. Two nominal electrode sizes were designed, 1.7 mm and 2.0 mm. The electrode spacing designed in CAD was 180 μm which resulted in an actual measured spacing between 137 and 144 μm in the inkjet-printed device. The measured average track widths were 149 and 181 μm for vertical and horizontal tracks, respectively, with 150 μm being the nominal size. Figure 5a shows one of the base plates of the PEDOT:PSS 4 × 4 array devices, while Figure 5b depicts the number identification for each of the electrodes in the 4 × 4 electrode array from 1 to 16. The middle electrodes were identified as electrode numbers 6, 7, 10, and 11.

**Table 1:** Nominal and Measured Size of the Inkjet-Printed PEDOT:PSS Electrodes in the 4 × 4 Electrode Array Device

		average measured size	
printed features	nominal size	horizontal (μm)	vertical (μm)
electrode size	1.7 mm	1744.2 ± 21.7	1716.6 ± 18.5
	2.0 mm	2060.5 ± 24.7	2040.8 ± 8.8
interelectrode spacing width	180 μm	144.2 ± 4.5	137.4 ± 7.1
track width	150 μm	181 ± 9.1	149.4 ± 5.4

**Figure 5:**
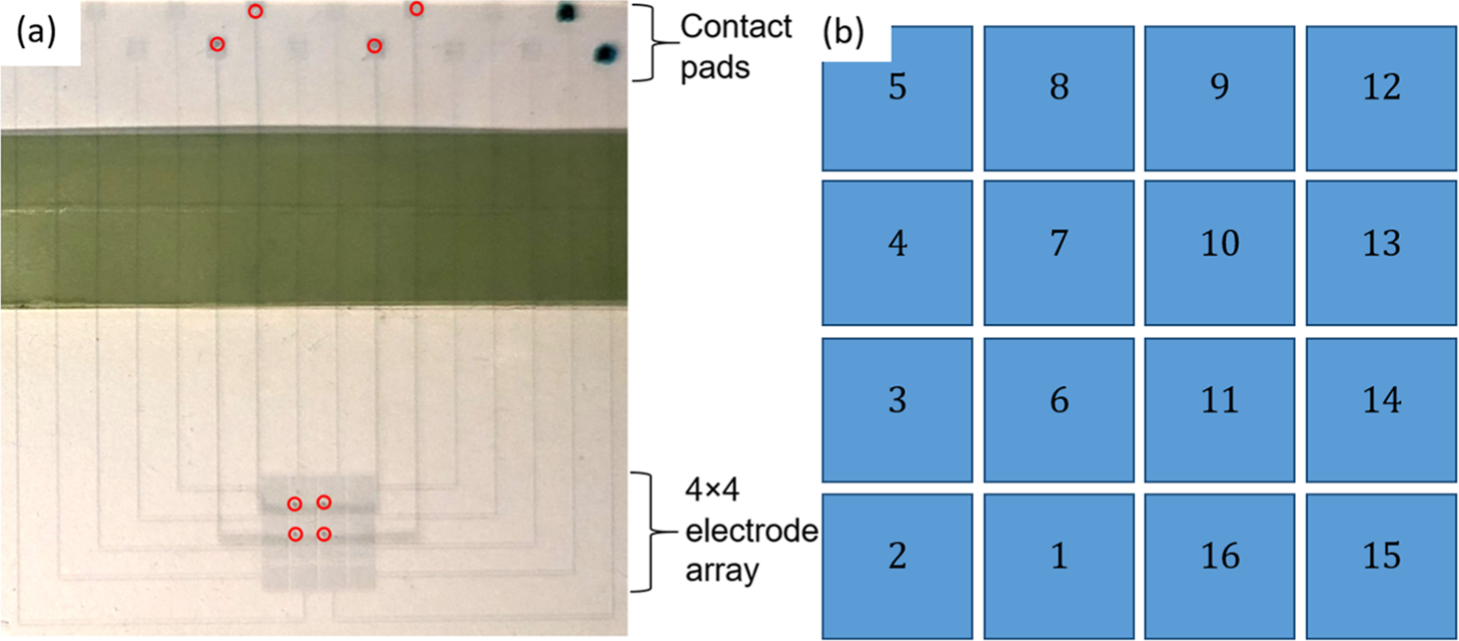
(a) One of the base plates of the PEDOT:PSS 4 × 4 array devices. The vias connecting the top and bottom surfaces of the plate are indicated in the red circles (b) the identification number for the 16 electrodes in the 4 × 4 electrode array device. (Photograph courtesy of E. Nadia. Copyright 2025).

The electrical resistance of inkjet-printed PEDOT:PSS conductive tracks (nominal dimensions: 10 mm in length and 250 μm in width) on a Melinex substrate was measured. The average resistance (mean ± standard deviation) was 9 ± 1 kΩ for tracks printed parallel to the printing direction and 16 ± 4 kΩ for tracks printed perpendicular to the printing direction. In addition, the minimum nominal track width that maintained electrical conductivity was 10 μm.

### Droplet Contact Angle with Applied Voltage

The electrowetting performance of the PEDOT:PSS-on-PET electrodes was evaluated by measuring the change in contact angle (CA) with varying applied voltage of a DI water droplet sitting on the electrode surface. The CA evolution on another type of electrode, ITO-on-glass was also measured for comparison. In Figure 6, the experimental data were plotted against the Young–Lippmann model, which predicts the CA change using the following equation:^46^

$$\cos\theta =\cos\theta_{0}+\frac{1}{2\gamma_{\mathrm{lg}}}cV^{2}$$

where θ is the contact angle at applied voltage, θ_0_ is the initial CA, γlg is the water–air interfacial tension, c is the capacitance per unit area, and V is the applied voltage. From Figure 6, the CA change induced by both types of electrode material followed the Young–Lippmann model until they diverged and remained at about the same contact angle of 73.3° and 74.0° for PEDOT:PSS-on-PET and ITO-on-glass electrodes, respectively, when the applied voltage reached 180 V. The unchanged CA is the saturation angle, where beyond this point the CA stops decreasing with the increasing voltage. Saturation angle is believed to occur due to the equilibrium reached between the electrostatic energy and the surface tension in the system.^47^ The 73.3° saturation angle for the PEDOT:PSS-on-PET is consistent with previously reported values of between 70° and 77° ^48,49^ where Cytop was also used as the hydrophobic surface. Both types of electrode, PEDOT:PSS-on-PET and ITO-on-glass demonstrated similar electrowetting response due to having the same electrowetting number $\left(\frac{\varepsilon_{o\varepsilon_{r}}V^{2}}{2d\gamma_{\mathrm{lg}}}\right)$ of 3.18 × 10^–5^ V^2^. The same thickness and type of dielectric (Parylene-C) and hydrophobic (Cytop) materials were used in both electrodes. This dimensionless number was calculated using the following values: permittivity of free space, ε_o_ = 8.85 × 10^–12^ F/m, Parylene-C dielectric constant, ε_r_ = 3.1, water–air interfacial energy, γ_lg_ = 72 mN/m, and dielectric thickness, *d* = 6 × 10^–6^ m.

**Figure 6:**
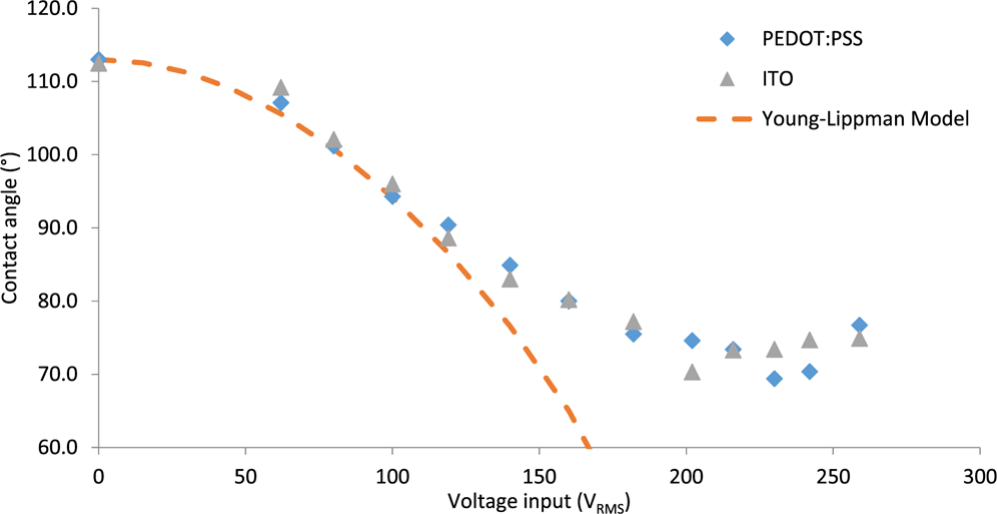
Change in DI water droplet contact angle with applied voltage for PEDOT:PSS and ITO electrodes. The results are compared with the contact angle predicted using the Young–Lippmann equation.

### Droplet Velocity with Applied Voltage

Experiments were conducted to evaluate the effect of voltage magnitude on droplet velocity. Apart from the PEDOT:PSS-on-PET device, the velocity of 2.5 μL of DI water droplet using 100 ms signal pulse with varying operating voltage was also measured on a chrome-on-glass device. The average velocity for a droplet to move across one electrode was measured by tracking the movement of the leading edge of the droplet using image analysis software. The velocity measurement was conducted only on regular control electrodes, the ones without vias laser-cut into them. On both types of electrode material, a minimum voltage of 75 V was required to actuate the droplet across one electrode. However, the droplet was not capable of reliably moving more than one or two electrodes across both electrode materials at this voltage. For the droplet to complete one full cycle reliably, covering the whole length of four electrodes back and forth, a minimum voltage of 120 V was required for the PEDOT:PSS-on-PET device, while 90 V was needed for the chrome-on-glass device.

Figure 7 shows the voltage-dependent average velocity of the PEDOT:PSS-on-PET and chrome-on-glass devices. At lower operating voltage (below 105 V), the PEDOT:PSS-on-PET has a higher average velocity than the chrome-on-glass device, but as the voltage was increased, the average velocity in the chrome-on-glass became higher than in the PEDOT:PSS-on-PET. The highest average velocities were 79.6 mm/s (at 195 V) and 93.8 mm/s (at 150 V) for the PEDOT:PSS-on-PET and chrome-on-glass devices, respectively. In both devices, the increase in average velocity with the operating voltage stopped once the voltage reached 150 V. For the PEDOT:PSS-on-PET device, the average droplet velocity exhibited a slight decrease beyond 150 V, but increased again as the voltage was raised to 195 V. The overall performance of this device is comparable to that reported by Pollack et al.,^4^ where an average droplet velocity of 30 mm/s was achieved using a 20 Hz signal and an electrowetting number of 1.34 at an operating voltage of 80 V.^36^ In comparison, the PEDOT:PSS device in this study yielded a projected average velocity of 27.4 mm/s at 80 V using a 100 ms actuation pulse.

**Figure 7:**
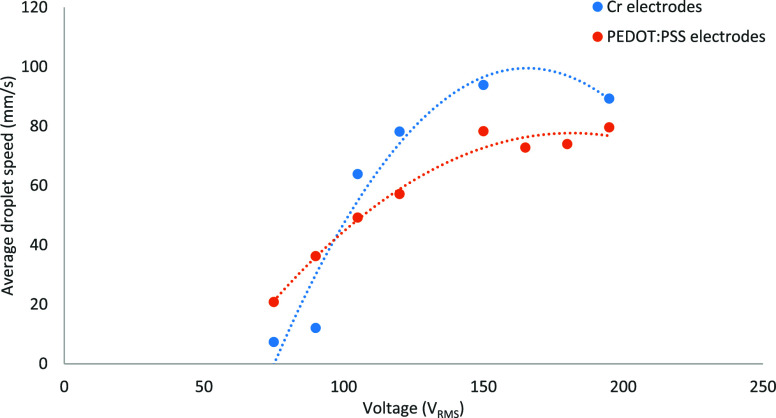
Average droplet velocity across one electrode for PEDOT:PSS-on-PET and chrome-on-glass devices.

### Droplet Transportation Across the 4 × 4 Electrode Array

Four electrode configurations were fabricated to evaluate their feasibility for transporting a 2.5 μL DI water droplet across the middle electrodes. The configurations varied in three parameters: electrode size (1.7 mm vs 2.0 mm), via position on the middle electrode (centre vs top-left corner), and via diameter (ranging from 110 to 225 μm). Table 2 summarises the results of droplet transport in the 4 × 4 array device for the four different configurations. All the droplet transportation sequences used 100 ms pulse signal.

**Table 2:**
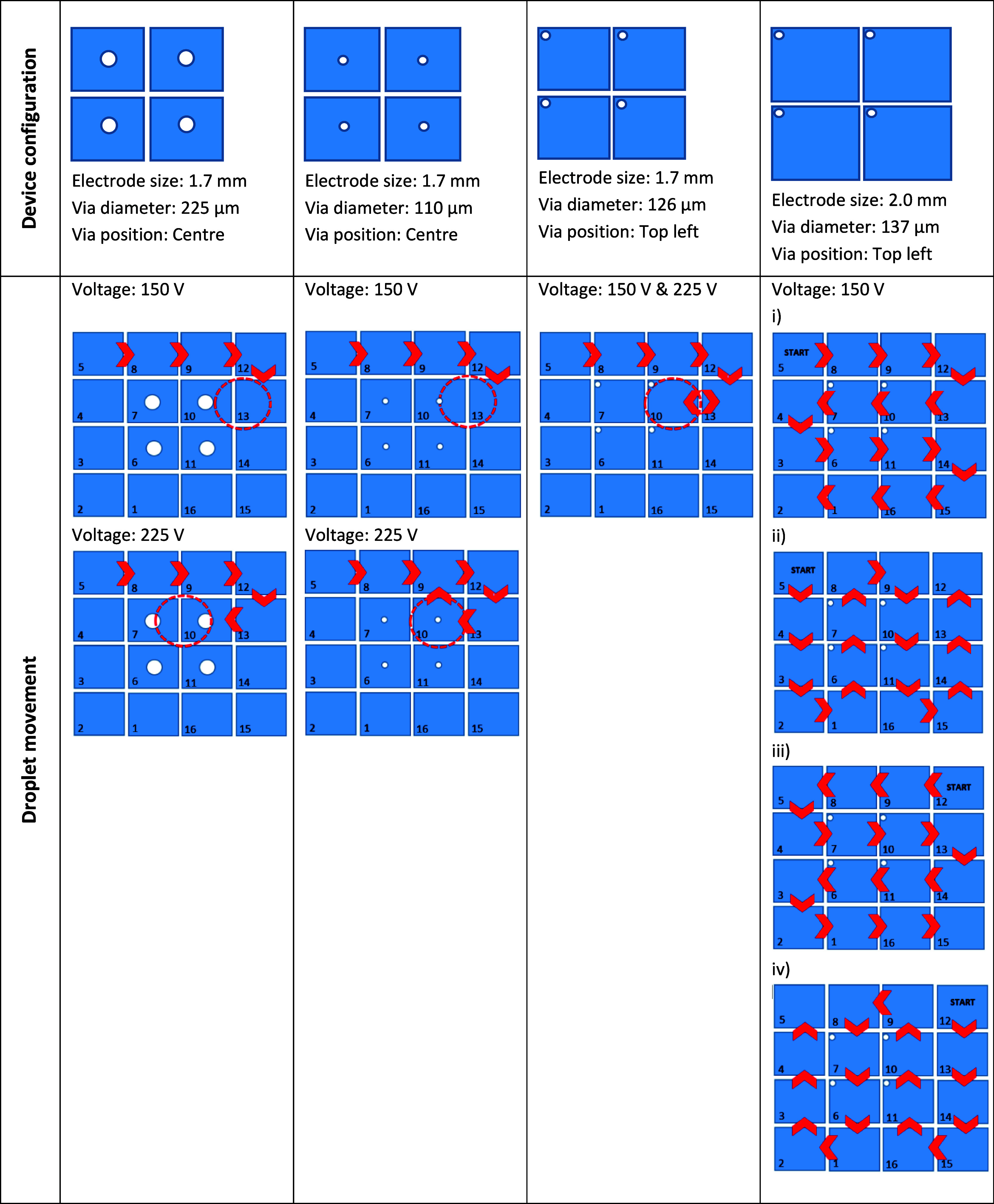
Droplet Movement in the 4 × 4 Array Device for Different Types of Configurations^a^

a The first row illustrates the design employed for the four middle electrodes, while the second row represents the droplet transportation across the 4 × 4 array. The red arrows indicate the direction of the droplet motion and the red dashed circles represent the position where the droplet became stuck in each device.

In the first configuration, the vias had the largest diameter of 225 μm, fabricated using a laser setting of 100% power and 8% speed, and were positioned at the centre of each 1.7 mm middle electrode. The electrode activation sequence followed the order 5–8–9–12–13–10–7 (as illustrated in Table 2), and an actuation voltage of 150 V was applied. Under these conditions, the droplet moved unimpeded up to the first middle electrode but got pinned between electrode 13 and middle electrode 10. When the actuation voltage was increased to 225 V, the droplet was able to advance past electrode 10, but subsequently became trapped between middle electrodes 10 and 7. Thus, while increasing the voltage from 150 to 225 V (2.25 × increased in the electrostatic force) was sufficient to overcome the barrier posed by the first middle electrode, it was insufficient to sustain motion across subsequent electrodes. The droplet remained trapped between two middle electrodes, which suggests that, in addition to diminishing the effective electrostatic force, the presence of a large via introduces a localised pinning effect originating from the via of the first middle electrode that impedes droplet motion.

The presence of a via, a nonconductive area on the electrode, also reduces the total capacitive energy required to drive the droplet to the middle electrode. Due to the diminishing electrostatic force in this region, the droplet contact line needs higher energy to move across a middle electrode. The electrostatic force, *F*_electrostatic_ generated on an activated electrode can be calculated using the following equation, where w is the width of the electrode^50^

$$F_{electrostatic}=\frac{w}{2}\frac{\varepsilon_{o}\varepsilon_{r}}{d}V^{2}$$

The exact mechanism underlying the pinning force introduced by the via is not investigated in this study; however, it is hypothesised to result from differences in wettability between the via region and the surrounding electrode surface, as proposed by Pit et al.^51^ According to their model, the pinning force, *F*_pin_ can be expressed as

$$F_{pin}=\gamma_{\mathrm{lg}}\left(\cos\theta_{philic}-\cos\theta_{phobic}\right)\cdot w_{p}$$

where *w*_p_ is the width of the via region, θ_philic_ and θ_phobic_ represent the Young’s contact angles on the surface of the via and the surrounding (deactivated) electrode, respectively. From this relation, the pinning force is expected to increase with the width of the via and the contrast in wettability between the hydrophilic and hydrophobic regions. This change in surface energy might be responsible for the observed droplet pinning. However, further investigation is required to confirm the underlying cause, particularly with respect to potential variations in local surface properties within the via region. One possible contributing factor is charge trapping within the via area, which may influence the apparent wettability and affect droplet dynamics.

To improve the droplet actuation across the middle electrodes, all subsequent configurations were made to have a smaller via diameter of below 140 μm. The second configuration has an electrode size of 1.7 mm with a via diameter of 110 μm, and the vias were positioned at the centre of each of the middle electrodes, similar to the first configuration. Here, the same problem was observed, whereby the droplet was trapped between electrode 13 and middle electrode 10 when 150 V operation voltage was used. However, when the voltage was increased to 225 V, the droplet was able to move onto electrode 10 but still could not overcome the pinning force to move onto the next middle electrode 7. However, the stuck droplet was able to move back to electrode 9 (next to electrode 10 and without a via) when the electrode activation sequence repeated itself. This indicates that the electrostatic force produced by an electrode without a via is large enough to overcome the pinning force created by a via with a smaller diameter. The smaller the via diameter, the smaller the pinning force trapping the droplet onto its surface, aligning with the model hypothesised based on differences in wettability between the via region and the surrounding electrode surface, proposed by Pit et al.^51^

In the third configuration, the position of the via on each middle electrode was modified from the centre to the top left corner. The electrode size remained at 1.7 mm, and the via diameter was comparable to that of the second configuration (126 μm). This adjustment allowed the droplet to move onto the middle electrode 10 without requiring the previously necessary high operating voltage of 225 V. It is postulated that relocating the via to the corner reduced its interference with the advancing contact line of the droplet, as the droplet does not encounter the via region immediately upon activation of the middle electrode. Despite this improvement, the droplet remained unable to move beyond the middle electrode 10. Increasing the voltage to 225 V did not overcome the barrier; instead, the droplet began oscillating between middle electrode 10 and electrode 13 during repeated activation cycles.

Finally, to successfully actuate the droplet across the middle electrode, the fourth configuration used a larger electrode than in the previous configurations. The electrode size used was 2 mm. Here, the droplet moved across all four middle electrodes from any direction using just 150 V. This demonstrates that using a larger electrode relative to the via size mitigates the effect of the pinning force, as the electrostatic force increases with the electrode size. Figures 8 and 9 show the sequence of a successful droplet transportation across the middle electrodes.

**Figure 8:**
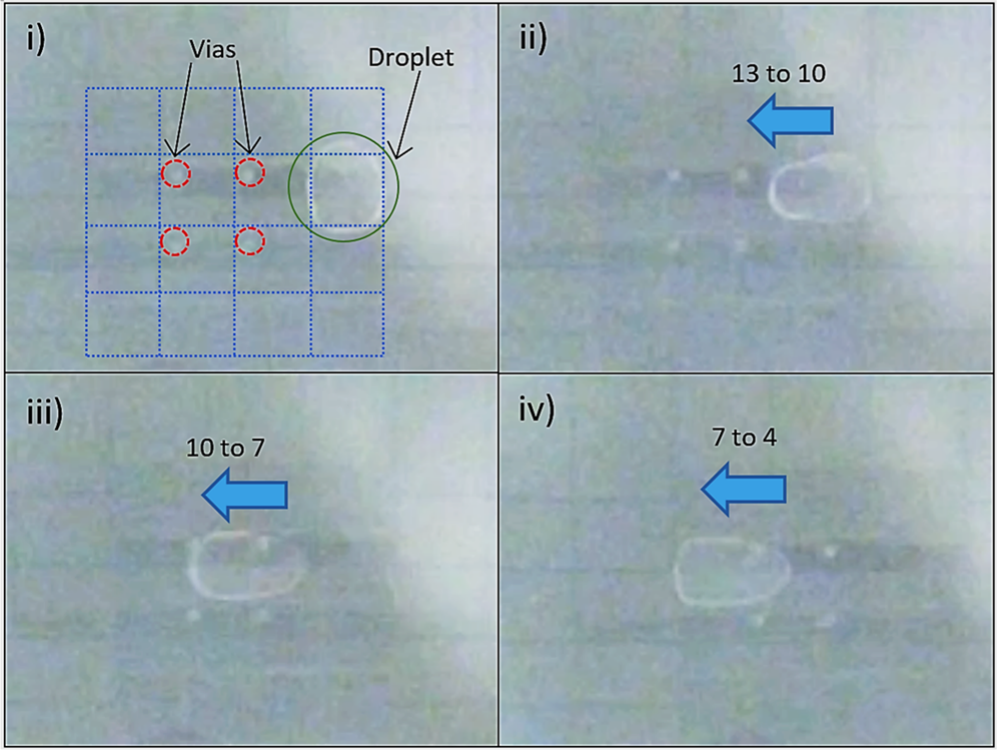
(i) Droplet movement across the electrodes (13 →10 →7→ 4) in the fourth configuration, where 10 and 7 are middle electrodes. The vias are positioned at the top left corner of each middle electrode. In the first image, the droplet is indicated in the green circle, the four vias are indicated inside the dashed red circles, and the blue dashed line outlines the 4 × 4 array. Initially, the droplet was positioned on electrode 13. (ii–iv) The second, third, and fourth images show the elongation of the droplet during its transportation as the next electrode was activated. The blue arrow indicates the direction of droplet movement. (Photograph courtesy of E. Nadia. Copyright 2025).

**Figure 9:**
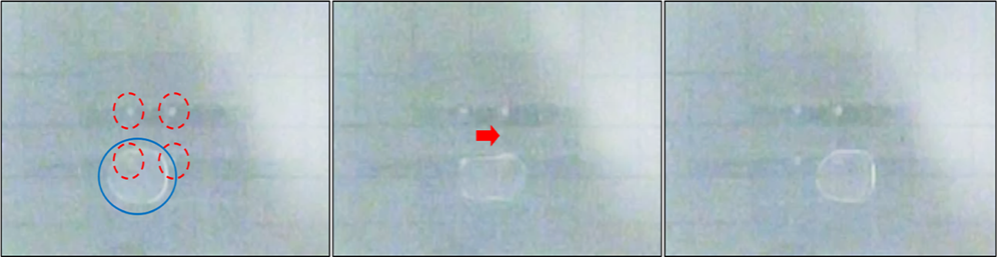
Droplet movement across one middle electrode, from electrode 6 to electrode 11. There are four middle electrodes with the vias positioned at the top left corner of each electrode. In the first image, the droplet is indicated in the blue circle while the four vias are indicated inside the dashed red circles. Initially, the droplet was positioned on electrode 6 (the bottom left electrode of the four middle electrodes). The second image shows the elongation of the droplet during its transportation to the next electrode as electrode 11 was activated. The red arrow indicates the direction of droplet movement. The third image shows the droplet in its final position, on electrode 11. (Photograph courtesy of E. Nadia. Copyright 2025).

In the fourth configuration, droplet motion could be initiated at an actuation voltage of 90 V, although a minimum of 105 V was required to move the droplet across two electrodes. Reliable transport across more than two electrodes necessitated an actuation voltage of at least 120 V. At this voltage, the droplet successfully completed only two full cycles across all 16 electrodes, including three additional electrodes required for returning to the starting point. When the actuation voltage was increased to 150 V, the droplet was able to complete up to 50 cycles of continuous movement, demonstrating the proof of concept for a low-cost, fully addressable DMF device.

## Conclusions

The aim of this study is to introduce a novel method for constructing a low-cost EWOD device with an electrode array larger than 3 × 3. Previously, large electrode array devices have been fabricated, but employing expensive techniques which are not readily available in most laboratories. The 4 × 4 device demonstrated at an initial level in this study used low-cost fabrication methods such as inkjet printing and laser cutting, which are more accessible than standard clean-room equipment. The materials used, conductive polymer PEDOT:PSS ink and PET Melinex substrate, are also inexpensive relative to the conventional materials, such as silver ink and semiconductor substrates.

The 4 × 4 electrode array device has successfully transported a water droplet across all 16 electrodes, demonstrating the feasibility of connecting different conductive layers through vias. The key to the connectivity between the different levels is the ink coverage on the inside surface of the vias. It has been demonstrated that silver ink does not provide adequate coverage on the inner surfaces of vias fabricated on the Melinex substrate, even after multiple printing passes. The PEDOT:PSS ink required only one printing pass on each surface to produce a successful electrical connection between the two levels.

Another challenge encountered during the fabrication process was determining an electrode and via design capable of reliably transporting droplets across an electrode surface containing a via. It was found that both the relative size of the via to the electrode and the via’s position on the electrode significantly affect the device’s ability to move droplets successfully. The introduction of a via creates heterogeneity on the actuating surface, which can impede droplet motion. Several configurations were evaluated, and the most effective design featured a large electrode size (2.0 mm) combined with a small via diameter (<140 μm). This finding suggests that a high electrode-to-via size ratio is critical for minimising the pinning effect introduced by the via while simultaneously enhancing the electrostatic force required to propel the droplet forward.

The 4 × 4 electrode array device offers a low-cost, accessible approach for constructing EWOD systems with a relatively large electrode array. A larger functional area enables higher throughput by allowing the execution of multiple parallel assays. Additionally, since the 4 × 4 array design utilises double-sided printing on the substrate, it facilitates the development of multilevel devices. The fabrication method proposed in this study is not only advantageous for EWOD applications but also has potential for broader use in electronic systems that require multilevel connectivity on a single substrate.

This capability not only reduces fabrication cost and complexity but also enables the realisation of novel electrode routing architectures that are difficult to achieve using conventional metal deposition methods. Consequently, inkjet-printed PEDOT:PSS electrodes provide an effective route to expand the number of addressable pads and enhance layout flexibility, thereby improving the overall configurability and scalability of EWOD platforms.
